# EDLMFC: an ensemble deep learning framework with multi-scale features combination for ncRNA–protein interaction prediction

**DOI:** 10.1186/s12859-021-04069-9

**Published:** 2021-03-19

**Authors:** Jingjing Wang, Yanpeng Zhao, Weikang Gong, Yang Liu, Mei Wang, Xiaoqian Huang, Jianjun Tan

**Affiliations:** Department of Biomedical Engineering, Faculty of Environment and Life, Beijing International Science and Technology Cooperation Base for Intelligent Physiological Measurement and Clinical Transformation, Beijing University of Technology, Beijing, 100124 China

**Keywords:** ncRNA–protein interactions, Multi-scale features combination, Conjoint k-mer, Ensemble deep learning, Independent test, ncRNA–protein networks

## Abstract

**Background:**

Non-coding RNA (ncRNA) and protein interactions play essential roles in various physiological and pathological processes. The experimental methods used for predicting ncRNA–protein interactions are time-consuming and labor-intensive. Therefore, there is an increasing demand for computational methods to accurately and efficiently predict ncRNA–protein interactions.

**Results:**

In this work, we presented an ensemble deep learning-based method, EDLMFC, to predict ncRNA–protein interactions using the combination of multi-scale features, including primary sequence features, secondary structure sequence features, and tertiary structure features. Conjoint k-mer was used to extract protein/ncRNA sequence features, integrating tertiary structure features, then fed into an ensemble deep learning model, which combined convolutional neural network (CNN) to learn dominating biological information with bi-directional long short-term memory network (BLSTM) to capture long-range dependencies among the features identified by the CNN. Compared with other state-of-the-art methods under five-fold cross-validation, EDLMFC shows the best performance with accuracy of 93.8%, 89.7%, and 86.1% on RPI1807, NPInter v2.0, and RPI488 datasets, respectively. The results of the independent test demonstrated that EDLMFC can effectively predict potential ncRNA–protein interactions from different organisms. Furtherly, EDLMFC is also shown to predict hub ncRNAs and proteins presented in ncRNA–protein networks of Mus musculus successfully.

**Conclusions:**

In general, our proposed method EDLMFC improved the accuracy of ncRNA–protein interaction predictions and anticipated providing some helpful guidance on ncRNA functions research. The source code of EDLMFC and the datasets used in this work are available at https://github.com/JingjingWang-87/EDLMFC.

**Supplementary Information:**

The online version contains supplementary material available at 10.1186/s12859-021-04069-9.

## Background

Genome sequencing in 2001 showed that only 2% of RNAs encode proteins, and 98% of RNAs do not code for proteins [1, 2], known as non-coding RNAs (ncRNAs). Studies have shown that ncRNAs are closely related to fundamental biological processes by interacting with RNA-binding proteins (RBPs) [3, 4], such as translation [5], splicing [6], chromatin remodeling [7], gene regulation [8], and many other life activities and functions [9–12]. In addition, ncRNAs implicate in cancer and other complex diseases [13–18]. Therefore, accurate prediction of ncRNA–protein interactions (ncRPIs) is crucial for understanding the regulatory function of ncRNAs and the pathogenesis of diseases.

High-throughput experimental techniques (RIP-Chip [19], HITS-CLIP [20], PAR-CLIP [21], etc.) and other experimental techniques of resolving complex structures (X-ray crystal diffraction (X-ray) [22], nuclear magnetic resonance (NMR) [23], electron cryo-microscopy (cryo-EM) [24], etc.) have been developed for revealing ncRPIs. However, experimental methods are time-consuming and labor-intensive [25]. Thus, there is a growing demand for the development of computational methods to predict ncRPIs.

Based on the features they used, computational methods to predict ncRPIs can be divided into two categories: sequence features as inputs and structure features as inputs. For sequence features based methods, lots of studies used machine learning or deep learning methods to learn features for predicting ncRPIs only based on the primary sequence. For instance, Muppirala et al. proposed a model named RPISeq, in which only primary sequence features were used, random forest (RF), or support vector machine (SVM) was used as classifiers to make predictions [26]. Pan et al. proposed a stacked ensemble model called IPMiner [27], learning primary sequence features from 3-mer and 4-mer frequency of protein and ncRNA, respectively. Then, Dai et al. designed a novel method, CFRP [28], put forward to generate complex features generated by non-linear transformations from the traditional k-mer features of ncRNA and protein primary sequences for characterizing ncRNA–protein interaction. RF was selected to reduce the dimensions of complex features and implement ncRNA–protein interaction (ncRPI) prediction tasks. Besides, Wang et al. utilized the deep convolutional neural network (CNN) to learn high-level features from the RNA and protein sequences, further feeding them into an extreme learning machine (ELM) for classification [29]. Furthermore, our group designed DM-RPIs, a classifier that integrated SVM, RF, and CNN to classify ncRPIs by learning the discriminative features from 3-mer and 4-mer frequency of proteins and ncRNAs, respectively [30]. In addition, LightGBM, rpiCOOL, RPIFSE, RPI-SAN, and LPI-CNNCP also made ncRPI predictions based on primary sequence [31–35].

For structure features based methods, besides sequence features, the often-used structure-derived features include secondary structure sequences, physicochemical properties, and others. Bellucci et al. proposed catRAPID [36, 37], which was based on the physiochemical properties of proteins and long non-coding RNAs (lncRNAs), including secondary structure, hydrogen bonding, and van der Waals propensities. Lu et al. proposed lncPro [38], using the same input features as Bellucci’s and fisher linear discriminant approach to implementing lncRNAs and proteins interaction predictions. Then, Suresh et al. proposed RPI-Pred [39], which combined the primary sequence and tertiary structure information of ncRNAs and proteins to predict ncRPIs. Lately, Peng et al. designed a hierarchical deep learning framework, RPITER [40], added more primary sequence information and sequence structure information by the improved conjoint triad feature coding method, which improved the classification performance of ncRPIs. Besides, Fan et al. considered pseudo nucleotide/amino acid composition and designed a novel computational method LPI-BLS by integrating logistic regression with five broad learning system classifiers [41], which performed a better classification performance than other state-of-the-art methods.

In the studies above, there are still few ones involving high-order 3D structural features. Our group found that the structural features play important roles in RNA-binding sites prediction, these structural characteristics reflect the properties around the binding sites, the clustering properties of the conserved interfacial residues, and the binding tendency [42]. We think structural features can also be used to predict ncRPIs. Furthermore, overwhelming majority of these relied on shallow machine learning techniques to implement classification tasks, such as fisher linear discriminant, RF, SVM, and logistic regression: lncPro employed fisher linear discriminant; RPI-seq employed RF and SVM; and LPI-BLS employed logistic regression. However, deep learning provides an approach to more effectively learn features from inputs and form high-level representations for more accurate prediction. One reason is that the increasing number of training samples can be derived from high-throughput sequencing techniques, which is highly beneficial for training deep learning models. The other is deep learning-based methods (especially CNN) that are powerful for analyzing spatial structure buried in data. And bi-directional long short-term memory network (BLSTM) is a widely used recurrent neural networks (RNN) with the memory cells, which can learn long dependency on the sequential data. Currently, CNN and BLSTM has been widely applied on computational biology and achieved superior performance in various biological sequence analysis problem [43], such as DNA function [44], RNA–protein binding sites [45] and protein–RNA binding preferences predictions [46].

Therefore, we proposed EDLMFC, a multi-scale features combination-based approach to predict ncRPIs through an ensemble deep learning model, which utilizes not only the primary sequence features of ncRNAs and proteins but also the structural features. These features are learned by layered networks, including CNN and BLSTM layers. Compared with the other three state-of-the-art methods, the comprehensive results demonstrate that EDLMFC has the best classification performance for ncRPI predictions.

## Results

### Performance comparison on EDLMFC with existing state-of-the-art methods

**Fig. 1:**
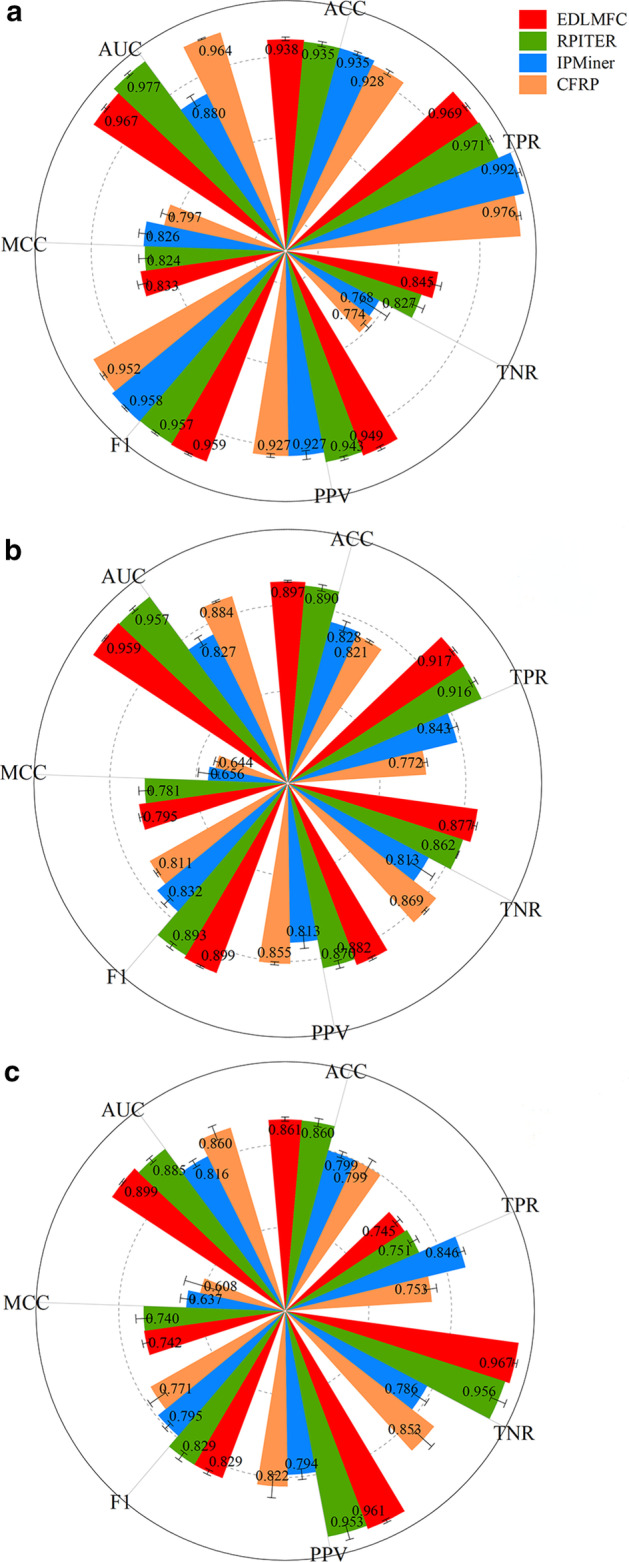
Performance comparison among different ncRPI prediction methods. **a** Performance comparison on RPI1807 dataset. **b** Performance comparison on NPInter v2.0 dataset. **c** Performance comparison on RPI488 dataset

To evaluate the performance of EDLMFC, we compared our method with the other three state-of-the-art methods. Since the work link of RPI-Pred was not available, and lncPro only provided the source code for the predictive model that has been trained on their dataset. Therefore, we chose RPITER, IPMiner, and CFRP to localize for comparation on RPI1807, NPInter v2.0, and RPI488 datasets under five-fold cross-validation (5CV), respectively. Seven performance metrics: accuracy (ACC), true positive rate (TPR), true negative rate (TNR), positive predictive value (PPV), F1-score (F1), Matthews correlation coefficient (MCC), and area under the curve (AUC) of the receiver operation characteristic (ROC), were employed to evaluate the above four methods comprehensively. The experimental results on RPI1807, NPInter v2.0, and RPI488 datasets are shown in Fig. 1a–c, respectively. And the detailed results are all listed in Table 1.

**Table 1:** Performance comparison between EDLMFC and other ncRPI prediction methods on RPI1807, NPInter v2.0, and RPI488

Dataset	Method	ACC (%)	TPR (%)	TNR (%)	PPV (%)	F1 (%)	MCC (%)	AUC (%)
RPI1807	EDLMC	**93.8 ± 0.3**	96.9 ± 0.3	**84.5 ± 0.9**	**94.9 ± 0.3**	**95.9 ± 0.2**	**83.3 ± 0.8**	96.7 ± 0.3
	RPITER	93.5 ± 0.4	97.1 ± 0.4	82.7 ± 1.1	94.3 ± 0.3	95.7 ± 0.2	82.4 ± 1.0	**97.7 ± 0.3**
	IPMinter	93.5 ± 0.3	**99.2 ± 0.4**	76.8 ± 2.4	92.7 ± 0.7	95.8 ± 0.2	82.6 ± 0.9	88.0 ± 1.0
	CFRP	92.8 ± 0.4	97.6 ± 0.4	77.4 ± 0.6	92.7 ± 0.3	95.2 ± 0.3	79.7 ± 0.9	96.4 ± 0.1
NPInter v2.0	EDLMFC	**89.7 ± 0.2**	**91.7 ± 0.4**	**87.7 ± 0.4**	**88.2 ± 0.3**	**89.9 ± 0.2**	**79.5 ± 0.4**	**95.9 ± 0.2**
	RPITER	89.0 ± 0.6	91.6 ± 0.6	86.2 ± 0.1	87.0 ± 0.8	89.3 ± 0.6	78.1 ± 1.2	95.7 ± 0.4
	IPMinter	82.8 ± 1.0	84.3 ± 0.9	81.3 ± 2.6	81.3 ± 1.3	83.2 ± 0.9	65.6 ± 2.0	82.7 ± 1.0
	CFRP	82.1 ± 0.3	77.2 ± 0.5	86.9 ± 0.3	85.5 ± 0.3	81.1 ± 0.3	64.4 ± 0.5	88.4 ± 0.2
RPI488	EDLMC	**86.1 ± 0.5**	74.5 ± 0.8	**96.7 ± 0.5**	**96.1 ± 0.4**	**82.9 ± 0.6**	**74.2 ± 0.9**	**89.9 ± 0.3**
	RPITER	86.0 ± 1.0	75.1 ± 1.1	95.6 ± 1.9	95.3 ± 1.8	82.9 ± 1.1	74.0 ± 1.9	88.5 ± 0.7
	IPMinter	79.9 ± 0.8	**84.6 ± 0.9**	78.6 ± 1.9	79.4 ± 1.3	79.5 ± 0.9	63.7 ± 1.6	81.6 ± 0.9
	CFRP	79.9 ± 2.0	75.3 ± 1.5	85.3 ± 2.7	82.2 ± 2.8	77.1 ± 2.3	60.8 ± 4.3	86.0 ± 1.8

The values in bold indicate this performance metric is the best among the four methods

The mathematical notation (±) represents standard deviation

From the Fig. 1a, EDLMFC achieves the highest ACC, TNR, PPV, F1, and MCC. As shown in Table 1, we can see that EDLMFC yielded an ACC of 93.8%, which is 0.3%, 0.3%, and 1.0% higher than that of RPITER, IPMiner, and CFRP, respectively. The standard deviation of ACC under 5CV is smaller than RPITER and CFRP. The TNR of EDLMFC is 84.5%, which is 1.8%, 7.7%, and 7.1% higher than that of RPITER, IPMiner, and CFRP, respectively. The PPV of EDLMFC is 94.9%, which is 0.6%, 2.2%, and 2.2% higher than that of RPITER, IPMiner, and CFRP, respectively. F1 of EDLMFC is 95.9%, which is 0.2%, 0.1% and 0.7% higher than that of RPITER, IPMiner and CFRP, respectively. MCC of EDLMFC is 83.3%, which is 0.9%, 0.7%, and 3.6% higher than that of RPITER, IPMiner, and CFRP, respectively. Although the TPR of EDLMFC is 2.3% lower than the IPMinter, the AUC is 1.0% lower than RPITER, EDLMFC method performs better than the two methods in general. Therefore, compared with the above three methods, our method EDLMFC has superior performance in predicting ncRPIs on RPI1807 dataset.

From the Fig. 1b, EDLMFC is superior to all the methods on seven performance metrics on NPInter v2.0 dataset. From the Fig. 1c, EDLMFC achieves the highest ACC, TNR, PPV, F1, MCC, and AUC on RPI488 dataset. It suggests that the method relied on integrated deep learning with a combination of multi-scale features presented in this work is an effective and efficient way to predict ncRPIs.

### Performance of EDLMFC in independent test

To further validate the ability of EDLMFC in distinguishing whether ncRNAs interact with proteins or not. We used the RPI1807 dataset to train our model and verified it on NPInter v2.0 dataset. There is no overlap between the two processed datasets. The processed NPInter v2.0 dataset contains 1943 interaction pairs, which can be divided into 6 organisms: Homo sapiens, Mus musculus, Saccharomyces cerevisiae, Caenorhabditis elegans, Drosophila melanogaster, and *Escherichia coli* with the number of interaction pairs of 740, 229, 693, 33, 46, and 202, respectively, which were tested by EDLMFC separately. As shown in Table 2, EDLMFC predicted the correct number of interacted pairs on the 6 organisms for 631, 217, 632, 31, 41, and 188, with ACC rates of 85%, 95%, 91%, 94%, 89%, and 93%, respectively. On the independent NPInter v2.0 dataset, we finally predicted the correct number of ncRNA–protein pairs to be 1740, with an overall ACC of 90%.

**Table 2:** Independent testing results of EDLMFC on six organisms from NPInter v2.0

Organism	Total ncRNA–protein pairs in NPInter v2.0	EDLMFC performance
Homo sapiens	740	631 (85%)
Mus musculus	229	217 (95%)
Saccharomyces cerevisiae	693	632 (91%)
Caenorhabditis elegans	33	31 (94%)
Drosophila melanogaster	46	41 (89%)
*Escherichia coli*	202	188 (93%)
Total	1943	1742 (90%)

### Analyses of different feature combination strategies

**Fig. 2:**
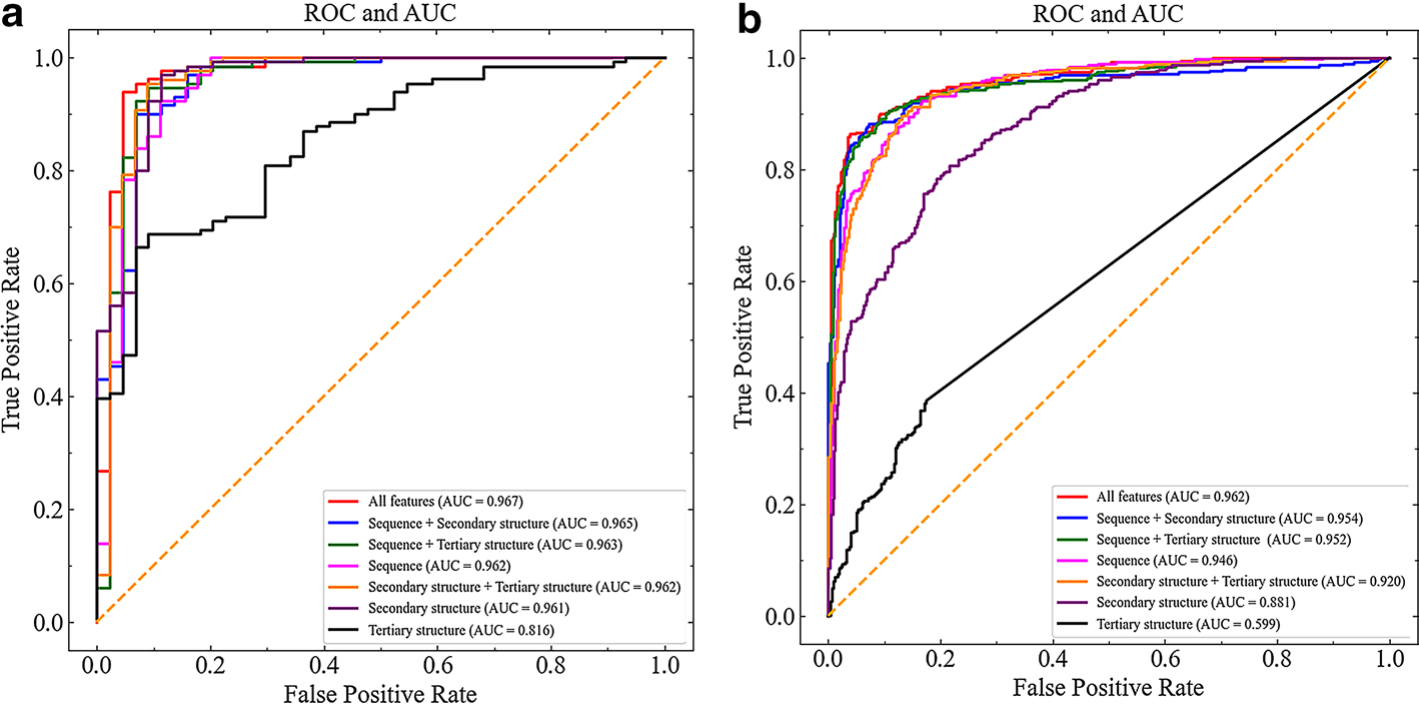
AUC comparison among different feature combination strategies. **a** AUC comparison on RPI1807 dataset. **b** AUC comparison on NPInter v2.0 dataset. The ROC curves of all feature, sequence together with secondary structure, sequence together with tertiary structure, only sequence, secondary structure together with tertiary structure, only secondary structure, and only tertiary structure as inputs were expressed in red, blue, green, magenta, orange, purple and black, respectively. The maximum AUC represents the best performance of the model

We adopted three kinds of feature of ncRNAs and proteins to construct EDLMFC model, including sequence features, secondary structure features, and tertiary structure features. To analyse the contributions of the three kinds of feature, seven different feature combinations: sequence, secondary structure, tertiary structure, sequence together with secondary structure, sequence together with tertiary structure, secondary structure together with tertiary structure, and all features were used as inputs to experiment the classification performance of the model. The ROC curves of seven different feature combinations as inputs tested on RPI1807 and NPInter v2.0 were shown in Fig. 2a and Fig. 2b, respectively. The results of seven performance metrics under 5CV are all listed in Table 3.

**Table 3:** Results under 5CV of different feature combinations considered on RPI1807 and NPInter v2.0

Dataset	Combinations of features	ACC (%)	TPR (%)	TNR (%)	PPV (%)	F1 (%)	MCC (%)	AUC (%)
RPI1807	Sequence	92.1 ± 1.3	94.5 ± 2.7	85.1 ± 2.9	94.9 ± 0.8	94.7 ± 0.9	79.5 ± 2.9	96.2 ± 0.8
	Secondary structure	92.8 ± 1.2	96.6 ± 1.9	81.5 ± 6.6	94.0 ± 2.1	95.2 ± 0.7	80.7 ± 3.5	96.1 ± 1.1
	Tertiary structure	72.9 ± 9.6	85.7 ± 21.7	28.7 ± 28.7	82.9 ± 6.9	79.7 ± 12.7	26.9 ± 21.5	81.6 ± 8.7
	Sequence + secondary structure	93.8 ± 0.6	96.6 ± 1.2	**85.5 ± 2.5**	**95.2 ± 0.8**	95.9 ± 0.4	83.5 ± 1.5	96.5 ± 0.8
	Sequence + tertiary structure	92.6 ± 1.6	95.1 ± 2.2	85.1 ± 2.9	95.0 ± 0.9	95.0 ± 1.1	80.5 ± 3.9	96.3 ± 0.8
	Secondary structure + tertiary structure	92.4 ± 0.4	96.5 ± 1.5	80.6 ± 5.6	93.7 ± 1.6	95.0 ± 0.2	79.7 ± 1.3	96.2 ± 0.9
	All features	**94.3 ± 0.2**	**97.4 ± 1.0**	85.1 ± 1.9	95.1 ± 0.6	**96.2 ± 0.2**	**84.7 ± 0.7**	**96.7 ± 0.8**
NPInter v2.0	Sequence	87.7 ± 0.8	89.7 ± 1.1	85.7 ± 2.4	86.3 ± 1.9	87.9 ± 0.7	75.5 ± 1.6	94.6 ± 0.3
	Secondary structure	78.8 ± 1.4	87.5 ± 1.4	70.1 ± 2.4	74.6 ± 1.6	80.5 ± 1.2	58.5 ± 2.7	88.1 ± 1.0
	Tertiary structure	54.7 ± 3.8	68.1 ± 27.0	41.4 ± 33.6	58.7 ± 8.7	56.3 ± 10.6	10.9 ± 8.7	59.9 ± 2.7
	Sequence + secondary structure	89.1 ± 0.9	91.2 ± 1.1	86.9 ± 1.5	87.5 ± 1.3	89.3 ± 0.8	78.3 ± 1.7	95.4 ± 0.3
	Sequence + tertiary structure	88.9 ± 0.8	91.1 ± 1.1	86.8 ± 1.3	87.3 ± 1.1	89.2 ± 0.7	77.9 ± 1.5	95.2 ± 0.5
	Secondary structure + tertiary structure	83.5 ± 1.0	88.6 ± 1.5	78.5 ± 2.6	80.5 ± 1.8	84.3 ± 0.7	67.5 ± 1.9	92.0 ± 0.4
	All features	**90.0 ± 0.7**	**92.2 ± 1.1**	**87.6 ± 0.9**	**88.2 ± 0.8**	**90.2 ± 0.7**	**80.0 ± 1.4**	**96.2 ± 0.3**

The values in bold indicate this performance metric is the best among the three methods

The mathematical notation (±) represents standard deviation

From the Fig. 2, on RPI1807 and NPInter v2.0 datasets, only secondary structure as input have a slightly lower AUC than only sequence as input and notably higher AUC than only tertiary structure as input. Thus, the sequence is the most important feature in ncRPIs; the following is the predicted secondary structure, and then is the tertiary structure. When any combination of two features was sent into the model, we find that its AUC value is higher than that of one of the two features. Moreover, the AUC value of the model is the highest when all features were entered. Therefore, we can conclude that all the features contain useful information, and at the same time, as inputs, they complement each other to give the model a better predictive performance.

### Application of EDLMFC for ncRNA–protein network construction

**Fig. 3:**
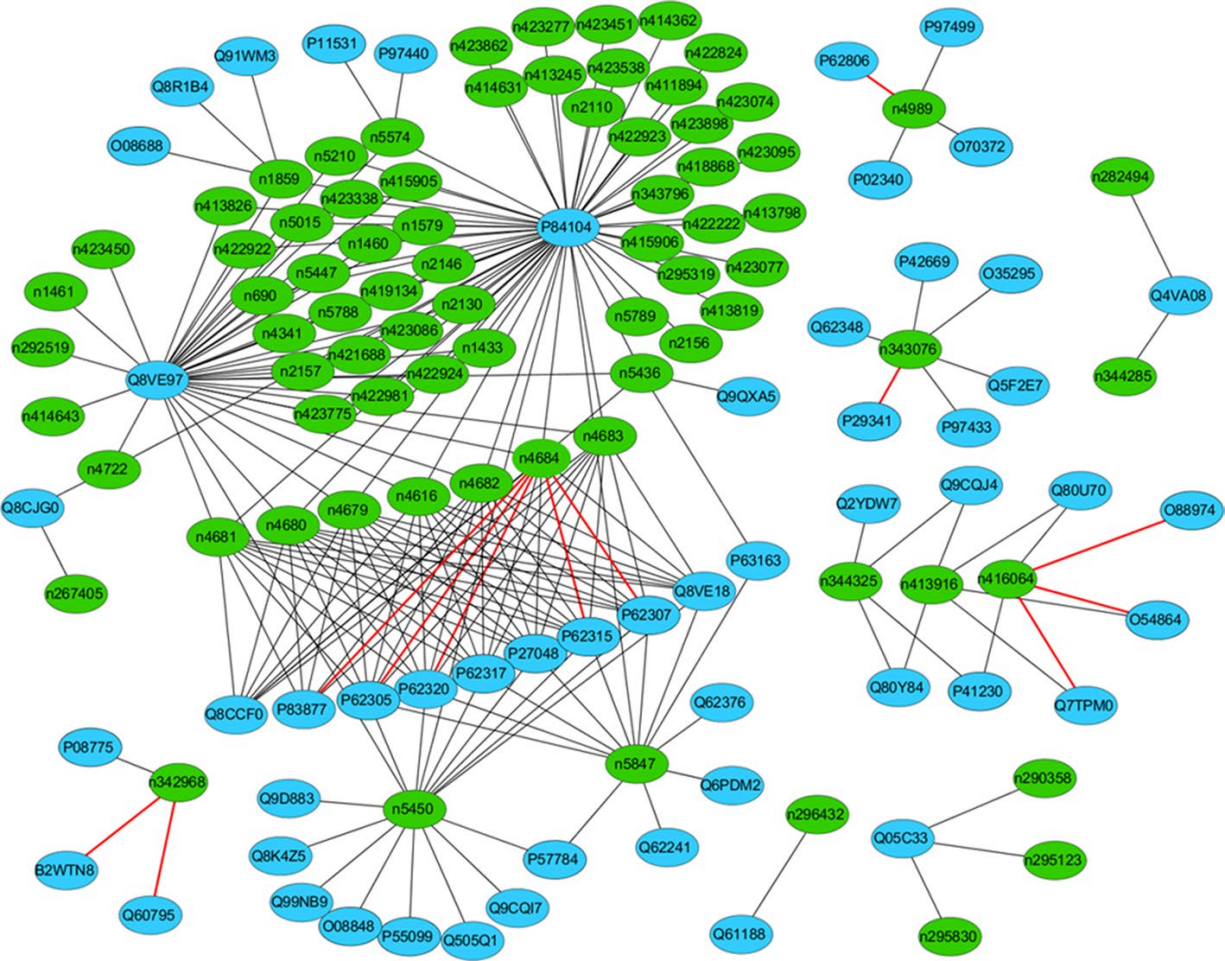
The Mus musculus networks constructed based on interaction pairs predicted by the EDLMFC. The oval nodes with green and blue represent the ncRNA and proteins, respectively. The gray and red edges indicate correctly and wrongly predicted ncRPIs, respectively

To visualize how many interactions have been correctly predicted, we further used the independent test results of EDLMFC to construct the ncRNA–protein networks. Here, we adopted a software named Cytoscape [47–49] for Mus musculus networks clustering. For Mus musculus in the NPInter v2.0 dataset, we correctly predicted the 217 of 229 interactions, the ACC up to 95%. As is shown in Fig. 3, we found that the ncRPIs of Mus musculus contain both hub proteins (a protein interacts with multiple RNAs) and hub ncRNAs (an RNA interacts with multiple proteins) [50]. P84104 and Q8VE97 hub proteins have the largest number of interactions and are both considered to be serine or arginine with rich splicing factor 3 [51]. Especially, P84104 hub protein is the splicing factor that specifically promotes exon-inclusion during alternative splicing. Interaction with YTHDC1, an RNA-binding protein that recognizes and binds N6-methyladenosine (m6A)-containing RNAs, promotes recruitment of SRSF3 to its mRNA-binding elements adjacent to m6A sites, leading to exon-inclusion during alternative splicing [52, 53]. Q8VE97 hub protein plays a role in alternative splice site selection during pre-mRNA splicing. Repressing the splicing of MAPT/Tau exon 10 as well [54]. Therefore, constructing ncRNA–protein networks help identify the important functions and pathways of key proteins and ncRNAs, which will facilitate various medical and pharmaceutical studies [55].

## Discussion

In this work, we proposed a multi-scale features combination-based computational method, EDLMFC, to predict ncRPIs through an ensemble deep learning combined CNN and BLSTM. Compared with the other three state-of-the-art methods on RPI1807, NPInter v2.0, and RPI488 datasets, comprehensive experimental results indicate our method EDLMFC has the best classification performance for ncRPI predictions. This is mainly because of the following reasons:

1. The multi-scale features were used, which includes not only sequence features information but also structural information. The results of different feature combinations show that sequence features are the most important, followed by secondary structure features and tertiary structure features. All features contain useful information, so the classification performance of the model was best when all features were used as input for prediction.
2. Using conjoint k-mer method to encode sequence features of ncRNAs and proteins, a variety of k-mer features are considered so that proteins and ncRNAs can be represented more accurately and comprehensively.
3. CNN was used to dig the hidden abstract high-level features of proteins and ncRNAs, then feeding into BLSTM to capture their long-range dependencies, and a three-layer fully-connected layer was employed to predict the ncRPIs.

Although EDLMFC achieves a better performance in ncRPI predictions, there are still some limits that need to be noticed. Like other deep learning-based approaches, it's like a black box that automatically learns the features of proteins and ncRNAs and makes predictions that we can't understand biologically. Besides, the method of ncRNA secondary structure prediction, SPOT-RNA, can only predict RNAs with a length of no more than 500 nucleotides. Therefore, our work mainly predicts the interaction between ncRNAs with a length of fewer than 500 nucleotides and proteins. In future work, we will consider designing more advanced neural network models to learn high-level abstract features with biological insights and choosing a more accurate prediction method of secondary structure to predict ncRPIs more accurately and efficiently.

## Conclusions

The prediction of ncRPIs contributes to understand the molecular mechanism within various fundamental biological processes and diseases. Many computational methods have been proposed for ncRPI predictions. However, only a small number of previous studies considered high-order structural features of ncRNAs and proteins, and overwhelming majority of them only used shallow machine learning to build classifiers for prediction. In this work, we presented a computational method based on CNN and BLSTM to predict ncRPIs through learning high-level abstract features from multi-scale features. To gain as much information of proteins and ncRNAs as possible, we employed not only primary sequence features, secondary structure sequence features but also tertiary structure features, and adopted a conjoint k-mer method to extract multiple-mers features by extending the range of k. Then, we adopted BLSTM to capture long-range dependencies between dominating features of ncRNAs/proteins learned by CNN, and send them to the full connection layer to predict whether they have the interaction relationship. Compared with the other three state-of-the-art methods under 5CV on RPI1807, NPInter v2.0, and RPI488 datasets, EDLMFC improved the performance with an increase of roughly 0.1%-7.7%. And the independent test between 6 organisms divided from NPInter v2.0 has an overall ACC of 90%, indicating that the ensemble deep learning framework can reveal and learn the high-level hidden information to improve prediction performance. Besides, according to the analyses of different feature combination strategies, we can conclude that all the features contain useful information. When multiple features were fed into the model, they complemented each other to make the model achieve a better prediction performance. In conclusion, EDLMFC method can be a useful tool for predicting unknown ncRPIs.

## Methods

### Benchmark datasets

Primary sequence data of paired samples, ncRNAs, and proteins in RPI1807, NPInter v2.0, and RPI488 were downloaded from the previous study [40]. RPI1807 has extracted the possible interaction pairs by parsing a nucleic acid database (NAD) that provides RNA protein complex and protein–RNA interface, consisting of 1078 RNA chains and 3131 protein chains in total [31]. In data preprocessing, the EMBOSS needle program has used to remove protein and RNA chains with high sequence similarity (cutoff ≥ 30%), then further distinguishing the atomic interactions with a distance threshold (cutoff = 3.40 Å), which was reasonable and sufficient to cover ‘strong’ and ‘moderate’ hydrogen bonds and energy-rich van der Waals contacts [56, 57]. It contains 1807 positive pairs and 1436 negative pairs after deleting the RNA sequences length of fewer than 15 nucleotides and the protein sequences of less than 25 amino acids. NPInter v2.0 was obtained from NPInter database, which documents functional interactions between noncoding RNAs (except tRNAs and rRNAs) and biomolecules (proteins, RNAs, and DNAs) verified by experiments [58]. In addition, as NPInter database only contains interaction (primarily physical interactions) pairs, and lack non-interaction pairs to work as negative samples in the training model, the same number of non-interaction pairs were generated by randomly pairing the ncRNAs and proteins in positive samples and further discarding similar known interaction pairs [26, 27] (a randomly generated pair R2–P2 was discarded if there has existed an interaction pair R1–P1 of P2 shared ≥ 40% sequence identity with P1 and R2 shared ≥ 80% sequence identity with R1). RPI488 is a lncRNA–protein interaction dataset, which was obtained from 18 ncRNA–protein complexes downloaded from the PDB database [27]. The atomic interactions were distinguished with a distance threshold (5 Å). CD-HIT tool [59] was used to remove protein and RNA chains with high sequence similarity (cut-off ≥ 90%). After redundancy removal, RPI488 dataset contains 488 lncRNA–protein pairs, including 243 interacting pairs and 245 non-interacting pairs.

Additionally, we used ncRNA secondary structure prediction method, SPOT-RNA, which was trained via RNAs with a maximum length of 500 nucleotides. Thus, ncRNAs with more than 500 nucleotides in primary sequence were deleted. ncRNAs-proteins paired samples of more than 500 nucleotides were further deleted based on the deleted ncRNA samples. Then, the protein primary sequences that were not paired with ncRNAs were deleted based on the deleted paired samples. Finally, RPI1807 contains 652 positive pairs and 221 negative pairs, NPInter v2.0 contains 1943 positive pairs and 1943 negative pairs. RPI488 contains 43 positive pairs and 233 negative pairs. The sample information of the original and processed set is shown in Table 4. Due to the large gap between the number of positive and negative samples in RPI488 dataset after processing. The negative samples were randomly divided into 5 groups to form 5 subsets with the positive samples. The average results of the 5 subsets were taken as the result of RPI488. The details of the 5 subsets are listed in Additional file 1: Table S1.

**Table 4:** The three original and processed ncRPI datasets used in this study

	Dataset	Positive pairs	Negative pairs	RNAs	Proteins
Original set	RPI1807	1807	1436	1078	3131
	NPInter v2.0	10,412	10,412	4636	449
	RPI488	243	245	25	247
Processed set	RPI1807	652	221	646	868
	NPInter v2.0	1943	1943	513	448
	RPI488	43	233	13	155

### Features extraction

#### SPOT-RNA based features

The secondary structure of ncRNA was predicted by SPOT-RNA [60, 61]. We localized their work by downloading it from https://github.com/jaswindersingh2/SPOT-RNA/. SPOT-RNA represented RSS with a macroscopic secondary structure, which is seven single character identifiers for the structure types of each nucleotide in the primary sequence. In this representation, S = stem, H = hairpin loop, M = multi-loop, I = internal loop, B = bulge, X = external loop, and E = end. Thus, each secondary structure sequence of ncRNAs can be represented by the seven-letter alphabet.

#### SPIDER3 based features

For protein secondary structure prediction, we localized SPIDER3 from the server http://www.sparks-lab.org/server/spider3/ [62], in which three classical protein secondary structures (α-helix, β-sheet, and coil) were used to represent each amino acid in the protein primary sequence. Besides, SPIDER3 also can be used to predict tertiary structures: solvent accessible surface area (ASA), contact number (CN), the upper half sphere exposure (HSEα-up), and the down half sphere exposure (HSEα-down) [62]. We calculated the average value of these tertiary structures for all amino acids in each protein sample.

#### Interface propensity

For interface propensity (IP) between a residue and nucleotide [63], we used an improved work by our team [63], which got the residue-nucleotide propensities (60 × 8) with secondary structure information of RNAs and proteins considered by scoring. Here, we calculated the average value of the binding preferences of all nucleotides to amino acids in a paired sample.

#### Sequence coding

To input ncRNA and protein sequences into deep learning or conventional machine learning models, the sequence data need to be encoded as numeric vectors. Most existing studies extracted ncRNA and protein sequence features by using a simple k-mer: 3-mer frequency feature for proteins and 4-mer frequency feature for ncRNAs [27, 30, 32, 35, 39]. For protein, 20 amino acids can be classified into seven groups based on their dipole moments and side-chain volume:${G}_{1}$={A, G, V}, ${G}_{2}$={I, L, F, P},${G}_{3}$={Y, M, T, S}, ${G}_{4}$={H, N, Q, W}, ${G}_{5}$={R, K}, ${G}_{6}$={D, E} and ${G}_{7}$={C} [39]. Then, each protein sequence can be represented by the seven-letter alphabet. Thus, a protein sequence can be represented as a numeric vector with 343 (${7}^{3}$) elements by calculating the 3-mer frequency. For ncRNA, using four ribonucleotides (A, U, G, C), an ncRNA sequence can be represented as a numeric vector of 256(${4}^{4}$) elements.

**Fig. 4:**
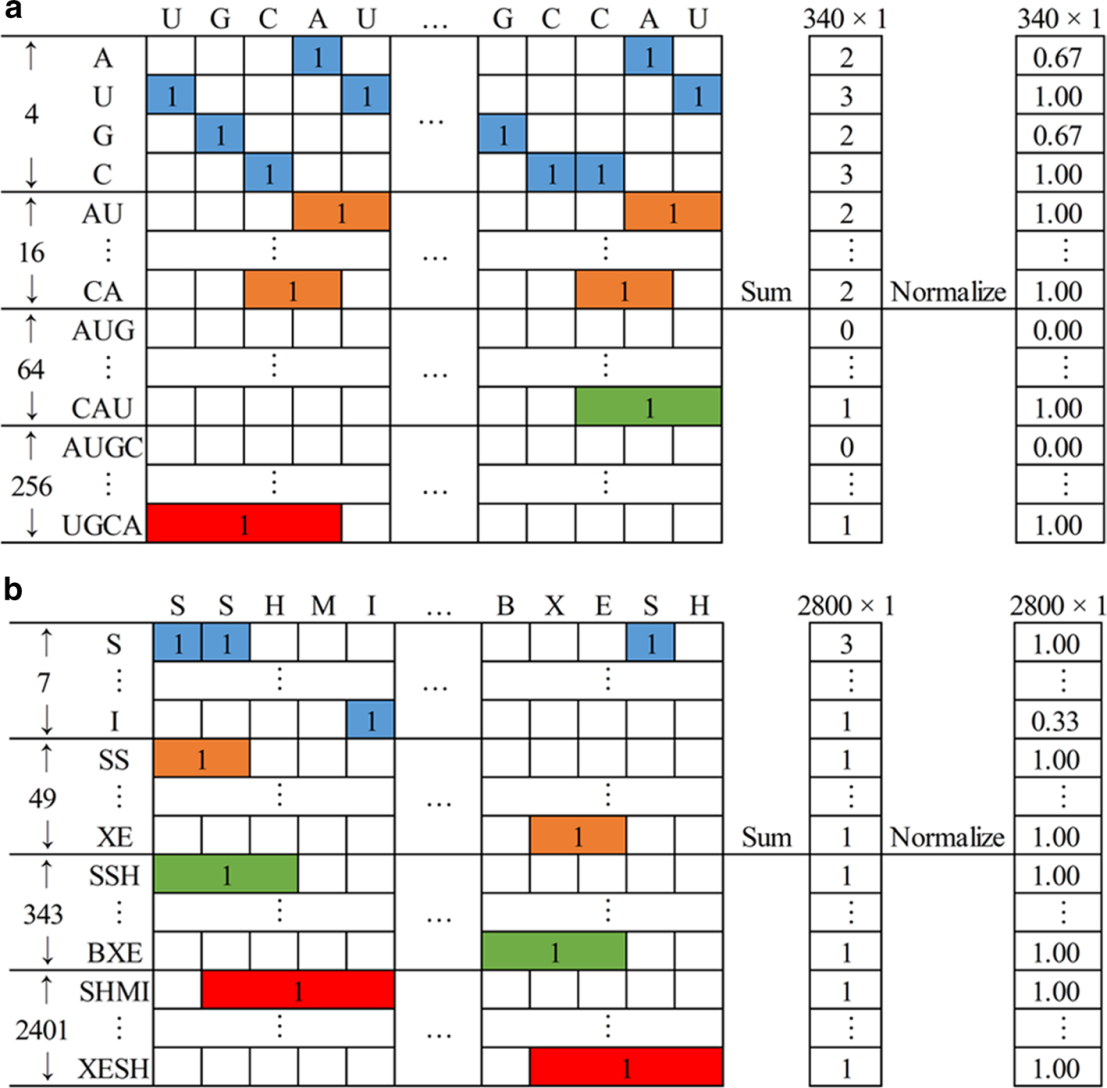
**a** The primary sequence of ncRNAs representation by conjoint k-mer. **b** The secondary structure sequence of ncRNAs representation by conjoint k-mer

We adopted a conjoint k-mer method to extract more feature information by extending the range of k to 1–4 in the k-mer frequency coding process for a ncRNA and 1–3 for a protein. That is to say, for ncRNA, we considered not only the 4-mer frequency information but also the 1-mer, 2-mer, and 3-mer. Similar to 4-mer, 3-mer of ncRNAs can be represented as a numeric vector with 64(${4}^{3}$) elements; 2-mer of ncRNAs can be represented as a numeric vector with 16(${4}^{2}$) elements; 1-mer of ncRNAs can be represented as a numeric vector with 4(${4}^{1}$) elements. As shown in Fig. 4a, the rows and columns are corresponding to all kinds of k-mer comprised of four ribonucleotides (A, U, G, C) and the primary sequence of each ncRNA. Then, a primary sequence of ncRNA can be represented by a binary matrix, which was then transformed into a numeric vector with 340 (${\sum }_{k=1}^{4}{4}^{k}$) elements by calculating each kind of k-mer frequency. Similar to Fig. 4b, using seven structure types (S, H, M, I, B, X, E), a secondary structure sequence of ncRNAs can be represented as a numeric vector with 2800 (${\sum }_{k=1}^{4}{7}^{k}$) elements. Therefore, integrating IP would produce the ncRNAs coding vector with 3141 (${\sum }_{k=1}^{4}{{4}^{k}+7}^{k}+1$) elements. For protein, we considered the 1-mer, 2-mer, and 3-mer frequency information, combining primary sequence represented by the reduced seven-letter alphabet, secondary structure sequence represented by three classical secondary structures (α-helix, β-sheet, and coil) with tertiary structures (IP, ASA, CN, HSEα-up and HSEα-down) would produce the proteins coding vector with 443 (${\sum }_{k=1}^{3}{{7}^{k}+3}^{k}+5$) elements.

### Performance metrics

We adopted 5CV to evaluate the performance of EDLMFC and other methods by seven widely used metrics. Due to the random effects of the training procedure, the 5CV was repeated 10 times. The average of the performance metrics predicted from the 10 times was used as the final prediction, and the 10 results of EDLMFC on the three datasets are listed in Additional file 1: Tables S2–S4. The formulas of ACC, TPR, TNR, PPV, F1, MCC, and AUC of the ROC are as follows:

1 $$ACC= \frac{TP+TN}{TP+TN+FP+FN}$$

2 $$TPR= \frac{TP}{TP+FN}$$

3 $$TNR= \frac{TN}{TN+FP}$$

4 $$PPV= \frac{TP}{TP+FP}$$

5 $$MCC= \frac{TP\times TN-FP\times FN}{\sqrt{(TP+FP)(TP+FN)(TN+FP)(TN+FN)}}$$

6 $$F1= \frac{2\times TPR\times PPV}{TPR+PPV}$$

where TP, FP, TN, and FN denote the number of true positive, false positive, true negative, and false negative, respectively. ACC reflects the ability of the classifier to discriminate against the whole sample. TPR reflects the ability to predict positive samples. TNR reflects the ability to predict negative samples. PPV represents the ability to discriminate positive samples that are actually positive samples. MCC reflects the classification performance of the classification model when the number of positive and negative samples are not balanced. F1 is a comprehensive index that considers TPR and PPV. And AUC is used to evaluate the performance of a classification model.

### Model design

**Fig. 5:**
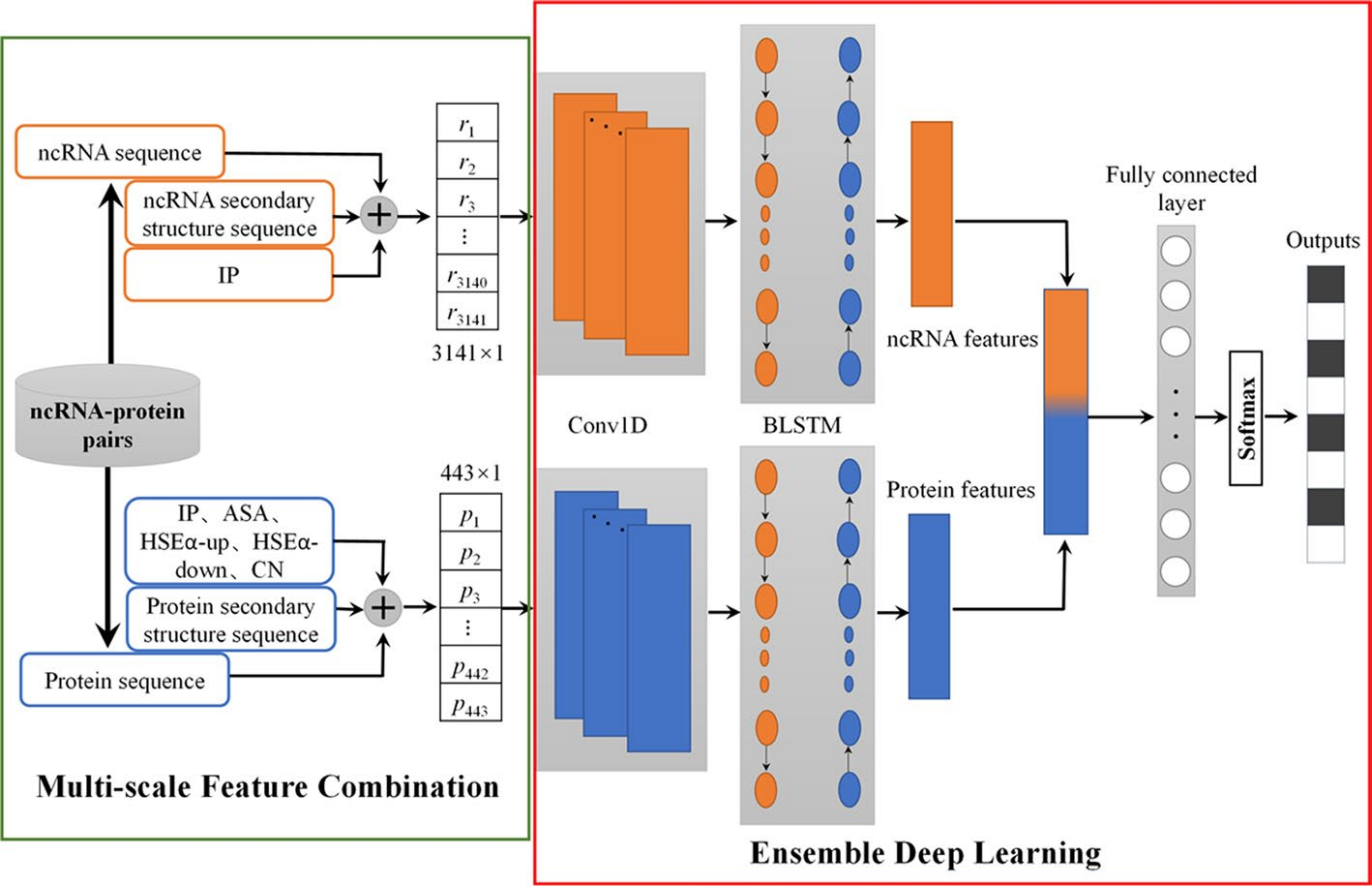
The workflow of the proposed EDLMFC

We adopted a conjoint k-mer to encode the primary sequence and secondary structure sequence features, merging IP and IP, ASA, HSEα-up, HSEα-down, CN for ncRNAs, and proteins, respectively, forming 3141 and 443-dimensional feature column vectors. Then the ensemble deep learning framework did the rest of the work automatically. Specifically, the two encoded feature column vectors of ncRNAs and proteins were separately fed into layered networks, including CNN and BLSTM layers. Then, a concatenated vector of the two outputs from the BLSTM layer was wired as the input of the fully connected layer. Finally, the ensemble module used the softmax activation function at the last layer to make binary predictions. The details of the proposed framework are shown in Fig. 5.

CNN consists of several layers, including the input layer, convolution layer, max-pooling layer, full connection layer, and output layer [64]. Among these, the convolution layer includes activation operation and the max-pooling layer includes batch normalization operation. In the convolution layer, assume that ${A}^{[l]}$ is the feature map of the $l\mathrm{th}$ layer, which can be described as:

7 $${A}^{[l]} = f\left({A}^{\left[l\right]} \otimes{W}^{\left[l\right]}+{b}^{\left[l\right]}\right)$$

where ${W}^{[l]}$ is the weight matrix of the convolution kernel of $l\mathrm{th}$ layer, operator $\otimes$ represents convolution operation, ${b}^{[l]}$ is the offset vector, and $f\left(x\right)$ is the activation function.

After convolution operation, a commonly used activation function rectified linear unit (ReLU) was applied to sparse the output of the convolution layer, which can be used to speed up the supervised train process and maintain the rate of convergence at a steady state to avoid the vanishing gradient problem [65]. Suppose that $\mathrm{ReLU}$ is the activating layer, its formula defined as:

8 $$ReLU = \left\{ {\begin{array}{*{20}l} {x,} \hfill & {if\, x > 0} \hfill \\ {0,} \hfill & {if \,x \le 0} \hfill \\ \end{array} } \right.$$

Followed by the convolution layer, the max-pooling layer was used to sample the feature graph according to certain rules to reduce the parameters and calculation while maintaining the main features. suppose that ${A}^{[l]}$ is the pooling layer, its formula is:

9 $${A}^{[l]} =sampling\left({A}^{\left[l-1\right]}\right)$$

After the max-pooling operation, batch normalization (BN) [66] operation was employed to reduce internal covariate shift and help train the designed deep network.

LSTM is a widely used RNN with the memory cells [67], which store information over an arbitrary time allowing the network to learn long dependencies in the sequential data. Three non-linear gating units (input, output and forget) control the information flow through the time steps. Each gate gets a similar input as the input neuron. Moreover, each gate has an activation function [68], which forward mechanism expressed by the following equation:10where *W*, *b* denote the weights and bias, respectively, $\sigma$ denotes the Logistic Sigmoid function, $*$ denotes pointwise multiplication, 
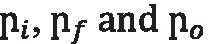
 represent the input gate, forget gate, and output gate, respectively. ${x}^{<\mathrm{t}>} is$ input data at current step t, ${a}^{<t-1>}$ is hidden state at previous step t − 1. ${c}^{<t-1>}$ is cell state at previous step t − 1,${c}^{<t>}$ is cell state at current step t,${a}^{<t>}$ is the hidden state at the current step t, which equal to the output ${y}^{<t>}$ at current.

We used the variant BLSTM, which consists of two parallel LSTMs: one input sequence forward and the other input sequence inverted [69], to capture long-range dependencies between high-level abstract features extracted from primary sequence, secondary structure sequence, and tertiary structure by CNN.

To predict ncRPIs effectively, we designed a training model based on a three-layer CNN combining BLSTM. Two similar ensemble neural network parts analyzed the ncRNA and protein input vectors separately, and two feature vectors were formed by using a one-layer fully-connected layer. Then, a three-layer fully-connected concatenated the two feature vectors as input and made the interaction prediction. The main parameters in the ensemble deep learning framework, including the number of layers, filter size, kernel size, learning rate, dropout rate, BLSTM hidden size, and fully-connection size, were tuned to maximize the MCC on a validation set randomly selected from the training set. For ensemble neural network of analyzing proteins, the values of the parameters are as follows: number of layers: 3; filter size: 45, 64, and 86; kernel size: 6, 6, and 6; and dropout rate: 0.2, 0.2, and 0.2; BLSTM hidden size: 45; fully-connection size: 64; For ensemble neural network of analyzing ncRNAs, the values of parameters are the same as the ones for analyzing proteins, except for kernel sizes which are 6, 5, and 5. In the end, the three-layer fully-connected with 128, 64, and 2 neurons, respectively, and the dropout with 0.25 and 0.3. Adam [70] and stochastic gradient descent (SGD) [71] were employed successively to train each part, among which Adam with a learning rate 0.001 first gave the module a quick converge and then SGD with a learning rate 0.005 was used to fine tune the module after. Besides, we used the back-propagation algorithm [72] to minimize the loss function of binary cross entropy, also used regularization [73] and early stopping [74] algorithms to avoid overfitting. Our model was implemented by the Keras2.2.5 library.

## Supplementary Information

**Additional file 1. Table S1**: The 5 subsets divided from the processed RPI488 dataset. **Table S2**: The results of EDLMFC under 5CV after running 10 times on RPI488 dataset. **Table S3**: The results of EDLMFC under 5CV after running 10 times on RPI1807 dataset. **Table S4**: The results of EDLMFC under 5CV after running 10 times on NPInter v2.0 dataset.

## Data Availability

The source code of EDLMFC and the datasets used in this work are available at https://github.com/JingjingWang-87/EDLMFC.

## Abbreviations

RBPs: RNA-binding proteins
ncRNA: Non-coding RNA
ncRPI: Non-coding RNA protein interaction
ncRNAs: Non-coding RNAs
ncRPIs: Non-coding RNA protein interactions
X-ray: X-ray crystal diffraction
NMR: Nuclear magnetic resonance
cryo-EM: Electron cryo-microscopy
CNN: Convolutional neural network
BLSTM: Bi-directional long short-term memory network
RNN: Recurrent neural networks
RSS: RNA secondary structures
IP: Interface propensity
ASA: Solvent accessible surface area
CN: Contact number
HSEα-up: A upper half sphere exposure
HSEα-down: A down half sphere exposure
RF: Random forest
SVM: Support vector machine
ELM: Extreme learning machine
lncRNAs: Long non-coding RNAs
NAD: Nucleic acid database
5CV: Five-fold cross-validation
ACC: Accuracy
TPR: True positive rate
TNR: True negative rate
PPV: Positive predictive value
F1: F1-score
MCC: Matthews correlation coefficient
AUC: Area under the receiver operating characteristic curve
ROC: Receiver operation characteristic
ReLU: Rectified linear unit
BN: Batch normalization
SGD: Stochastic gradient descent
