# Supplementary material for: EDLMFC: an ensemble deep learning framework with multi-scale features combination for ncRNA–protein interaction prediction
Source: BMC Bioinformatics. 2021 Mar 19;22:133. doi: 10.1186/s12859-021-04069-9 (PMC7980572; doi:10.1186/s12859-021-04069-9)
Supplement: Supplementary file 1 — Additional file 1. Table S1: The 5 subsets divided from the processed RPI488 dataset. Table S2: The results of EDLMFC under 5CV after running 10 times on RPI488 dataset. Table S3: The results of EDLMFC under 5CV after running 10 times on RPI1807 dataset. Table S4: The results of EDLMFC under 5CV after running 10 times on NPInter v2.0 dataset. [file 12859_2021_4069_MOESM1_ESM.docx]

**Table S1.** The 5 subsets divided from the processed RPI488 dataset.

|  | **Positive Pairs** | **Negative Pairs** |
| --- | --- | --- |
| **Subset1** | 43 | 46 |
| **Subset2** | 43 | 46 |
| **Subset3** | 43 | 47 |
| **Subset4** | 43 | 47 |
| **Subset5** | 43 | 47 |

**Table S2.** The results of EDLMFC under 5CV after running 10 times on RPI488 dataset.

|  | **ACC(%)** | **TPR(%)** | **TNR(%)** | **PPV(%)** | **F1(%)** | **MCC(%)** | **AUC(%)** |
| --- | --- | --- | --- | --- | --- | --- | --- |
| **1** | 85.7 | 74.7 | 95.7 | 94.7 | 82.8 | 73.0 | 90.5 |
| **2** | 87.1 | 76.6 | 96.6 | 96.0 | 84.3 | 75.9 | 90.0 |
| **3** | 86.6 | 75.6 | 96.5 | 96.0 | 83.4 | 75.1 | 90.0 |
| **4** | 86.6 | 74.1 | 97.9 | 97.1 | 83.4 | 75.1 | 90.2 |
| **5** | 85.6 | 73.6 | 96.6 | 95.8 | 81.8 | 73.4 | 89.8 |
| **6** | 85.9 | 73.7 | 97.0 | 96.4 | 82.5 | 74.0 | 89.3 |
| **7** | 85.3 | 73.3 | 96.2 | 95.7 | 82.1 | 72.7 | 89.3 |
| **8** | 85.9 | 74.7 | 96.2 | 96.0 | 82.9 | 74.2 | 90.2 |
| **9** | 85.7 | 73.7 | 96.5 | 95.9 | 82.5 | 73.5 | 90.1 |
| **10** | 86.8 | 74.9 | 97.4 | 96.9 | 83.6 | 75.4 | 89.5 |
| **Average** | **86.1±0.5** | **74.5±0.8** | **96.7±0.5** | **96.1±0.4** | **82.9±0.6** | **74.2±0.9** | **89.9±0.3** |

The mathematical notation (±) represents standard deviation.

**Table S3.** The results of EDLMFC under 5CV after running 10 times on RPI1807 dataset.

|  | **ACC(%)** | **TPR(%)** | **TNR(%)** | **PPV(%)** | **F1(%)** | **MCC(%)** | **AUC(%)** |
| --- | --- | --- | --- | --- | --- | --- | --- |
| **1** | 93.8 | 96.5 | 86.0 | 95.3 | 95.9 | 83.6 | 96.8 |
| **2** | 93.4 | 97.2 | 81.9 | 94.1 | 95.6 | 82.1 | 96.3 |
| **3** | 93.7 | 97.1 | 83.7 | 94.6 | 95.8 | 83.0 | 96.7 |
| **4** | 93.5 | 96.6 | 84.2 | 94.7 | 95.7 | 82.6 | 96.8 |
| **5** | 93.5 | 96.8 | 83.7 | 94.6 | 95.7 | 82.5 | 96.1 |
| **6** | 93.4 | 96.0 | 85.5 | 95.2 | 95.6 | 82.5 | 96.5 |
| **7** | 94.3 | 97.4 | 85.1 | 95.1 | 96.2 | 84.7 | 96.7 |
| **8** | 94.0 | 97.1 | 85.1 | 95.0 | 96.1 | 84.0 | 97.4 |
| **9** | 93.9 | 96.9 | 85.1 | 95.0 | 96.0 | 83.7 | 97.2 |
| **10** | 94.3 | 97.4 | 85.1 | 95.1 | 96.2 | 84.7 | 96.7 |
| **Average** | **93.8±0.3** | **96.9±0.3** | **84.5±0.9** | **94.9±0.3** | **95.9±0.2** | **83.3±0.8** | **96.7±0.3** |

The mathematical notation (±) represents standard deviation.

**Table S4.** The results of EDLMFC under 5CV after running 10 times on NPInter v2.0 dataset.

|  | **ACC(%)** | **TPR(%)** | **TNR(%)** | **PPV(%)** | **F1(%)** | **MCC(%)** | **AUC(%)** |
| --- | --- | --- | --- | --- | --- | --- | --- |
| **1** | 89.8 | 91.4 | 88.2 | 88.5 | 89.9 | 79.6 | 95.6 |
| **2** | 90.0 | 91.6 | 88.4 | 88.8 | 90.1 | 80.0 | 95.9 |
| **3** | 89.2 | 91.3 | 87.2 | 87.7 | 89.5 | 78.6 | 95.6 |
| **4** | 89.4 | 91.6 | 87.2 | 87.8 | 89.6 | 78.9 | 95.7 |
| **5** | 89.6 | 92.0 | 87.3 | 87.9 | 89.9 | 79.4 | 95.9 |
| **6** | 89.8 | 92.4 | 87.2 | 87.9 | 90.1 | 79.7 | 96.1 |
| **7** | 89.4 | 91.6 | 87.3 | 87.8 | 89.6 | 78.9 | 95.5 |
| **8** | 89.8 | 91.0 | 88.5 | 88.8 | 89.9 | 79.6 | 96.0 |
| **9** | 89.9 | 92.2 | 87.6 | 88.2 | 90.2 | 80.0 | 96.2 |
| **10** | 90.0 | 92.2 | 87.6 | 88.2 | 90.2 | 80.0 | 96.2 |
| **Average** | **89.7±0.2** | **91.7±0.4** | **87.7±0.4** | **88.2±0.3** | **89.9±0.2** | **79.5±0.4** | **95.9±0.2** |

The mathematical notation (±) represents standard deviation.
